# Complete Genome Sequences of 47 Environmental Isolates of Escherichia coli

**DOI:** 10.1128/MRA.00222-20

**Published:** 2020-09-17

**Authors:** Georgia Breckell, Olin K. Silander

**Affiliations:** a School of Natural and Computational Sciences, Massey University, Auckland, New Zealand; University of Maryland School of Medicine

## Abstract

Escherichia coli is commonly considered a host-associated bacterium. However, there is evidence that some strains occupy environmental (non-host-associated) niches. Here, we report the complete genomes of 47 Escherichia coli environmental isolates. These will be useful for understanding the dynamics of plasmids, phages, and other repetitive genetic elements.

## ANNOUNCEMENT

Escherichia coli has historically been considered a host-associated bacterium, although recent evidence suggests that many strains may persist and grow in the environment, and in some cases this may be the primary niche (1–4). E. coli is also well known for its prolific horizontal gene transfer (3, 5). To understand the rates of gene transfer, especially of mobile genetic elements (which are often repetitive in nature), complete genomes are required. To achieve this, we carried out whole-genome sequencing and assembly for 47 environmental strains of E. coli isolated from the shore of the St. Louis River in Minnesota, near Lake Superior (6).

We grew all strains in LB medium and isolated genomic DNA using either the Promega Wizard kit or phenol-chloroform extraction (7). All strains were sequenced using both the Oxford Nanopore Technologies (ONT) and Illumina sequencing platforms. Illumina data were obtained from MicrobesNG with in-house quality control (adapter trimming with Trimmomatic v0.30, with a sliding window quality score cutoff value of Q15) using DNA extracted with the Promega Wizard kit. We prepared ONT sequencing libraries using the rapid barcoding kit (SQK-RBK004) and ran all libraries on R9.4 flow cells, multiplexing between 6 and 12 strains on each flow cell. We performed base calling using Guppy v2.3.7. We obtained at least 250 Mbp of sequence data for all strains except one (Table 1), with a median of 1,002 Mbp per strain (interquartile range [IQR], 670 Mbp to 1,296 Mbp). For all strains with more than 500 Mbp of sequence data, we used Filtlong v0.2.0 (https://github.com/rrwick/Filtlong) to retain only 500 Mbp in total, prioritizing read quality over length with the following parameters: min length set to 1,000, mean q weight set to 10, and split set to 500. The filtered read sets had a median read *N*_50_ value of 17.4 kbp (IQR, 13.4 kbp to 20.5 kbp). We also obtained at least 30-fold coverage of 2 × 250-bp paired-end Illumina reads for each genome.

**TABLE 1:** Genome statistics for all 47 assemblies

Strain ID^*a*^	Sample date (yr-mo-day)	Location	No. of Illumina reads	ONT extraction method^*b*^	Total ONT sequence size (bp)	No. of ONT reads	ONT read *N*_50_ (bp)	Filtered ONT read *N*_50_(bp)^*c*^	Chromosome length (bp)^*d*^	Circular chromosome^*e*^	Genome length (bp)^*f*^	Total no. of contigs	rRNA orientation^*g*^	Read accession no.	Assembly accession no.
SC468	2005-8-15	Upshore	1,288,488	Phenol-CHCl_3_	1,402,515,335	34,337	7,164	14,815	4,426,017	Yes	4,426,017	1	Standard	SAMEA6595239	GCA_902825195
SC457	2005-8-15	Upshore	603,943	Phenol-CHCl_3_	465,520,117	51,693	9,209	10,280	4,555,909	Yes	4,555,909	1	Standard	SAMEA6595235	GCA_902810185
SC455	2005-8-15	Upshore	819,314	Phenol-CHCl_3_	1,033,029,654	30,634	10,560	17,536	4,655,420	Yes	4,655,420	1	Standard	SAMEA6595233	GCA_902810345
SC434	2005-8-15	Waterline	618,526	Phenol-CHCl_3_	849,800,865	33,880	10,113	16,056	4,658,197	Yes	4,658,197	1	Standard	SAMEA6595225	GCA_902810315
SC477	2005-9-19	Surface water	1,045,031	Phenol-CHCl_3_	843,013,946	44,613	7,856	12,549	4,658,510	Yes	4,658,510	1	Standard	SAMEA6595243	GCA_902810395
SC316	2005-6-15	Surface water	3,409,700	Promega	43,287,859	39,445	11,807	13,365	4,663,327	Yes	4,681,204	3	Standard	SAMEA6595204	GCA_902809975
SC467	2005-8-15	Upshore	922,562	Phenol-CHCl_3_	1,003,216,478	38,336	8,521	13,963	4,715,938	Yes	4,715,938	1	Standard	SAMEA6595238	GCA_902825205
SC423	2005-8-15	Sediment	849,233	Phenol-CHCl_3_	274,260,727	18,522	16,188	18,066	4,716,885	Yes	4,716,885	1	Standard	SAMEA6595220	GCA_902810325
SC465	2005-8-15	Upshore	1,577,380	Promega and phenol-CHCl_3_	1,386,503,691	35,047	9,489	15,195	4,722,586	Yes	4,785,019	2	Standard	SAMEA6595237	GCA_902810145
SC452	2005-8-15	Upshore	417,390	Phenol-CHCl_3_	2,207,184,721	16,959	11,909	30,889	4,723,951	Yes	4,755,166	2	Standard	SAMEA6595230	GCA_902810195
SC431	2005-8-15	Waterline	722,122	Phenol-CHCl_3_	1,774,332,186	25,144	3,326	21,138	4,727,732	Yes	4,866,254	2	Standard	SAMEA6595223	GCA_902810235
SC475	2005-9-19	Surface water	413,920	Phenol-CHCl_3_	907,422,842	36,085	8,263	14,996	4,729,401	Yes	4,729,401	1	Standard	SAMEA6595241	GCA_902810385
SC492	2005-9-19	Surface water	544,889	Phenol-CHCl_3_	718,894,150	30,095	13,767	18,501	4,736,913	Yes	4,736,913	1	Standard	SAMEA6595248	GCA_902810375
SC480	2005-9-19	Surface water	614,748	Phenol-CHCl_3_	1,009,767,146	25,686	12,892	20,491	4,741,504	Yes	4,741,504	1	Standard	SAMEA6595245	GCA_902810405
SC476	2005-9-19	Surface water	661,043	Promega and phenol-CHCl_3_	1,467,536,905	33,435	6,509	15,758	4,747,946	Yes	4,747,946	1	Standard	SAMEA6595242	GCA_902810415
SC479	2005-9-19	Surface water	483,518	Phenol-CHCl_3_	468,589,667	41,638	11,800	13,367	4,762,128	Yes	4,913,012	3	Standard	SAMEA6595244	GCA_902810125
SC392	2005-8-24	Upshore	870,803	Promega	366,289,283	44,990	8,646	9,714	4,770,015	Yes	4,783,281	2	Standard	SAMEA6595208	GCA_902810015
SC312	2005-6-15	Surface water	3,582,617	Promega	678,059,117	32,708	13,222	17,765	4,775,485	Yes	4,878,778	3	Standard	SAMEA6595203	GCA_902810065
SC386	2005-8-18	Upshore	1,917,879	Promega	989,915,389	4,259	7,170	12,507	4,778,381	Yes	5,088,094	5	Standard	SAMEA6595207	GCA_902810055
SC456	2005-8-15	Upshore	350,577	Phenol-CHCl_3_	120,521,609	14,793	8,716	9,946	4,790,285	Yes	4,885,342	2	Standard	SAMEA6595234	GCA_902810135
SC487	2005-9-19	Surface water	1,289,213	Phenol-CHCl_3_	651,178,805	34,634	13,099	17,546	4,794,586	Yes	4,794,586	1	Standard	SAMEA6595246	GCA_902810365
SC433	2005-8-15	Waterline	566,913	Phenol-CHCl_3_	660,660,048	25,781	17,312	23,362	4,797,429	Yes	4,961,214	2	Standard	SAMEA6595224	GCA_902810215
SC429	2005-8-15	Waterline	1,110,243	Promega and phenol-CHCl_3_	1,081,882,331	28,668	9,271	18,780	4,797,468	Yes	4,961,244	2	Standard	SAMEA6595221	GCA_902810175
SC430	2005-8-15	Waterline	2,171,974	Promega and phenol-CHCl_3_	1,749,627,499	27,343	7,478	19,159	4,797,499	Yes	4,961,283	2	Standard	SAMEA6595222	GCA_902810205
SC397	2005-8-15	Surface water	1,485,195	Promega	1,001,724,414	28,040	13,643	19,677	4,858,696	No	5,067,247	3	Standard	SAMEA6595209	GCA_902809965
SC411	2005-8-15	Surface water	565,996	Phenol-CHCl_3_	1,360,250,706	25,134	11,224	20,582	4,859,344	Yes	5,068,109	3	Standard	SAMEA6595216	GCA_902810035
SC419	2005-8-15	Sediment	793,357	Phenol-CHCl_3_	1,421,369,578	35,942	6,732	14,684	4,859,796	Yes	4,916,116	2	Standard	SAMEA6595218	GCA_902810255
SC364	2005-7-27	Surface water	9,348,468	Promega	1,077,384,666	26,426	13,126	20,928	4,860,085	Yes	5,063,812	2	Standard	SAMEA6595205	GCA_902810045
SC453	2005-8-15	Upshore	522,987	Promega and phenol-CHCl_3_	1,231,172,695	50,059	7,010	11,072	4,863,138	No	5,308,239	4	Standard	SAMEA6595231	GCA_902810175
SC307	2005-6-15	Surface water	2,670,106	Promega	1,216,450,680	26,661	10,595	19,865	4,892,106	Yes	5,221,106	5	Standard	SAMEA6596823	GCA_902809955
SC400	2005-8-15	Surface water	9,809,258	Promega	1,138,079,055	21,521	16,543	25,690	4,924,724	Yes	5,065,688	2	Standard	SAMEA6595210	GCA_902809905
SC489	2005-9-19	Surface water	779,700	Phenol-CHCl_3_	530,260,839	73,424	5,806	7,779	4,929,025	No	5,008,168	4	Standard	SAMEA6595247	GCA_902810115
SC469	2005-9-19	Surface water	399,522	Phenol-CHCl_3_	723,447,743	40,512	9,772	13,766	4,940,057	Yes	5,129,818	3	Standard	SAMEA6595240	GCA_902810165
SC402	2005-8-15	Surface water	3,305,579	Promega	1,103,528,651	25,927	14,104	20,875	4,944,324	Yes	5,085,287	2	Standard	SAMEA6595211	GCA_902810085
SC406	2005-8-15	Surface water	700,028	Promega and phenol-CHCl_3_	2,471,779,990	25,820	8,152	19,613	4,958,102	Yes	4,958,102	1	Standard	SAMEA6595213	GCA_902810285
SC454	2005-8-15	Upshore	1,653,791	Phenol-CHCl_3_	1,010,953,794	43,107	7,741	13,039	4,982,834	Yes	4,988,386	2	Alternative	SAMEA6595232	GCA_902810105
SC441	2005-8-15	Waterline	738,159	Phenol-CHCl_3_	988,454,259	46,048	6,225	11,435	4,986,040	Yes	5,022,479	2	Alternative	SAMEA6595226	GCA_902810245
SC446	2005-8-15	Waterline	1,037,860	Phenol-CHCl_3_	451,143,119	35,499	13,071	14,580	4,986,746	Yes	4,997,068	2	Alternative	SAMEA6595229	GCA_902810265
SC445	2005-8-15	Waterline	490,093	Phenol-CHCl_3_	260,701,609	14,322	19,183	21,437	4,987,469	Yes	5,033,261	2	Alternative	SAMEA6595228	GCA_902810225
SC443	2005-8-15	Waterline	841,953	Phenol-CHCl_3_	1,027,640,114	31,687	9,897	17,265	4,999,711	No	5,021,047	2	Alternative	SAMEA6595227	GCA_902810185
SC410	2005-8-15	Surface water	436,740	Phenol-CHCl_3_	981,338,093	25,375	10,873	22,404	5,001,654	Yes	5,008,324	2	Standard	SAMEA6595215	GCA_902809925
SC422	2005-8-15	Sediment	514,475	Phenol-CHCl_3_	611,712,027	98,763	4,900	5,623	5,003,951	Yes	5,003,951	1	Standard	SAMEA6595219	GCA_902810275
SC464	2005-8-15	Upshore	874,611	Promega and phenol-CHCl_3_	3,086,301,299	18,303	8,993	27,897	5,023,622	Yes	5,137,983	2	Standard	SAMEA6595236	GCA_902810155
SC407	2005-8-15	Surface water	1,260,500	Promega and phenol-CHCl_3_	2,703,637,269	24,419	7,660	20,853	5,088,866	Yes	5,088,866	1	Standard	SAMEA6595214	GCA_902810295
SC403	2005-8-15	Surface water	1,257,142	Promega	765,980,798	33,276	12,830	17,205	5,089,116	Yes	5,145,436	2	Standard	SAMEA6595212	GCA_902809945
SC368	2005-7-31	Surface water	4,766,180	Promega	910,447,798	48,174	6,454	11,026	5,101,998	Yes	5,101,998	1	Standard	SAMEA6595206	GCA_902810305
SC418	2005-8-15	Sediment	665,222	Promega and phenol-CHCl_3_	2,677,454,945	24,282	7,752	21,140	5,222,289	Yes	5,222,289	1	Standard	SAMEA6595217	GCA_902810335

a Strain identification (ID) and sampling information were taken from reference 6. Strains are sorted by chromosome length.

b Phenol-CHCl_3_ indicates that phenol-chloroform extraction was used; Promega indicates that the Promega Wizard DNA extraction kit was used.

c Filtered read *N*_50_ indicates the *N*_50_ value after Filtlong (https://github.com/rrwick/filtlong) was used to retain only 500 Mbp from each strain.

d Chromosome length indicates the length of the longest contig, assumed to be the chromosome.

e Circular chromosome indicates whether the chromosome is a single circular contig.

f Genome length indicates the sum of the lengths of all contigs.

g rRNA orientation indicates the orientation of the seven ribosomal operons in E. coli, as assessed by socru (11).

We used the long-read assembler Flye v2.4.2 (8) for genome assembly. We polished the assemblies using four rounds of long-read polishing with Pilon v1.23 (9), followed by two rounds of short-read polishing with Racon v1.3.2 with the following parameter changes: gap penalty increased to −8, match score increased to 8, and mismatch score increased to −6 (10). The contigs were left as linear if not circularized by Flye, with no reorientation. We confirmed the structural accuracy of each genome using socru v2.1.7 (11) to assess the order and orientation of the seven rRNA operons (Table 1). In all cases, the rRNA operons were found in standard or known orientations, supporting the structural accuracy of these genomes. All software was run using default parameters, unless otherwise specified.

The genomes range in length from 4.4 Mbp to 5.2 Mbp. Under the assumption that any nonchromosomal contigs are plasmids, 17 isolates contained no plasmids (i.e., only one chromosomal contig), 21 isolates contained a single plasmid, and 10 isolates contained multiple plasmids (Table 1). The 47 complete genomes produced in this study provide a resource for insight into environmental adaptation and the genome dynamics of repetitive mobile genetic elements in E. coli.

### Data availability.

The complete sequences and reads for these isolates have been deposited in the ENA database, and the accession numbers are listed in Table 1.
